# Insect rearing protocols in forensic entomology: Benefits from collective rearing of larvae in a carrion beetle *Necrodes littoralis* L. (Silphidae)

**DOI:** 10.1371/journal.pone.0260680

**Published:** 2021-12-01

**Authors:** Joanna Gruszka, Szymon Matuszewski

**Affiliations:** 1 Laboratory of Criminalistics, Adam Mickiewicz University, Poznań, Poland; 2 Wielkopolska Center for Advanced Technologies, Adam Mickiewicz University, Poznań, Poland; 3 Department of Animal Taxonomy and Ecology, Adam Mickiewicz University, Poznań, Poland; North Carolina State University, UNITED STATES

## Abstract

Forensic entomologists frequently use a developmental method to estimate a post-mortem interval (PMI). Such estimates are based usually on the blow fly larvae or puparia. Data on their development is obtained by rearing them in colonies. In the case of beetles, which can be also useful for PMI estimation, development data is frequently collected by rearing them individually. However, some carrion beetles are gregarious, for instance, *Necrodes littoralis* (Linnaeus, 1758) (Silphidae). We compared mortality, rate of development and body size of emerged adult beetles reared individually and in aggregations. Mortality was much higher for beetles reared individually, particularly at low temperatures. The rearing protocol affected the time of immature development and the size of adult insects. Individually reared specimens developed much longer at 16°C, whereas at 20°C and 26°C development times of individually reared beetles were slightly shorter. Significant differences in the body size were observed only at 16°C; beetles that developed in aggregations were larger at this temperature. These findings demonstrate that aggregating is particularly beneficial for larvae of *N*. *littoralis* at low temperatures, where it largely reduces mortality and facilitates growth. Moreover, these results indicate that in forensic entomology the protocol of individual rearing is unsuitable for gregarious beetles, as it produces reference developmental data of low quality.

## Introduction

The developmental method is one of the tools used in forensic entomology to determine post-mortem interval (PMI), which is the time that elapsed from death to disclosure of the body. The foundation for its use is reference data from developmental studies carried out on species of carrion insects. The laboratory protocol for such studies is to monitor insect development under several constant temperatures. Then, the data collected is used to derive developmental models, for instance, the thermal summation model that uses accumulated degree days (ADD, the accumulation of heat above the threshold temperature over a certain number of days) [1]. ADD necessary for the development of insects found on a cadaver can be used to estimate the development interval or the minimum post mortem interval (PMI_min_), which is the minimum time that elapsed from death [1,2]. Insects in forensic entomology are bred under a variety of conditions: individually or in aggregations consisting of various numbers of larvae. A rearing protocol may affect the quality of the data and in effect the development models, especially when insects are reared under different conditions than those occurring in nature. A literature review, outlined below, shows that there are no standards in forensic entomology in regard to the size of insect cultures that are used to collect development data.

Most frequently, blow flies are used for the estimation of PMI_min_ [3]. Data on larval development is obtained by rearing blow fly larvae in aggregations consisting of tens or hundreds of individuals. In the development studies on *Chrysomya megacephala* (Fabricius, 1794) (Diptera: Calliphoridae), for instance, larvae were reared in aggregations consisting of 10 insects [4]. Larger aggregations were used in the research on *Phormia regina* (Meigen, 1826) (Diptera: Calliphoridae), in case of which there were 400 larvae per aggregation [5]. However, the most common size of the aggregation was 100 larvae [6–8].

In the case of beetles, rearing protocols frequently consisted in monitoring individual insects (both larvae and pupae) that developed in separate containers. For instance, *Creophilus maxillosus* (Linnaeus, 1758) (Coleoptera: Staphylinidae) larvae were reared separately in 80-ml (1^st^ instar larvae) and 120-ml (3^rd^ instar larvae) containers [9]. In the study of *Dermestes undulatus* Brahm, 1790 (Coleoptera: Dermestidae) and *D*. *frischii* Kugelann, 1792 (Coleoptera: Dermestidae), each larva was kept in a different jar [10]. In studies on the development of *Thanatophilus micans* (Fabricius 1794) and *T*. *capensis* (Wiedemann, 1821) (Coleoptera: Silphidae), individual larvae were kept in Petri dishes [11,12]. Similarly, in studies of *D*. *maculatus* De Geer, 1774 (Coleoptera: Dermestidae) larvae were reared in separate containers [13].

There were also studies in which beetle larvae were reared in aggregations. Their size, however, never exceeded 30 larvae. *Thanatophilus sinuatus* (Fabricius, 1775) (Coleoptera: Silphidae) were reared in Petri dishes in groups of up to 5 larvae [14]. In the case of *Oxelytrum discicolle* (Brullé, 1836) (Coleoptera: Silphidae), the number of larvae per jar varied between 10 and 30 [15]. In studies of *Omosita colon* (Linnaeus, 1758) (Coleoptera: Nitidulidae), there were about 22 larvae per Petri dish [16]. *Dermestes* species were bred in aggregations of 10 larvae [17]. Similarly, in the case of *Necrobia rufipes* (De Geer, 1775) (Coleoptera: Cleridae), there were 10 larvae per Petri dish [18].

Laboratory protocols, in which larvae are reared individually, are suitable for predators, species prone to cannibalism or solitary insects, but may not be suitable for gregarious species that in natural conditions feed in larval aggregations. Feeding in aggregations can be beneficial for insects due to many reasons [19]. This behaviour can make foraging more efficient, lowering mortality and decreasing the duration of development [20]. Moreover, it affects the temperature experienced by the larvae. Blow fly and *Necrodes* larvae can raise the temperature inside the feeding aggregation and thus minimize the negative effects of fluctuating ambient air temperatures [21,22]. Estimating the age of a species that naturally feed in aggregations, by using the data from a protocol in which larvae were reared individually, may lower the accuracy of estimation. Because the aggregation increases foraging efficiency and raises the temperature in the feeding microenvironment [22], individual rearing conditions may lower survival and extend the development. In consequence, by using such a biased data, insect age and eventually the minimum PMI may be overestimated. Depending on the density of larvae in an aggregation, massing can also affect the size of adult insects [23,24]. It may be important, as the size of adult *N*. *littoralis* may be used to improve the accuracy of PMI estimation [25].

*Necrodes littoralis* (Linnaeus, 1758) (Coleoptera: Silphidae: Silphinae) is a forensically important, necrophagous beetle [26–28], which is widely distributed in the Palearctic region. Females of *Necrodes* lay eggs in large batches into the soil near the corpse [29]. After hatching larvae go through three larval instars [30]. Postfeeding larvae bury into the soil to form a pupal chamber, where they pupate and eventually reach the adult stage [29]. In natural conditions these beetles feed on large cadavers, where they usually form large aggregations of larvae [31] and under favourable conditions can independently drive active decay [26]. A recent study showed that temperature plays an important role in the formation and maintenance of larval aggregations, as larvae reacted to changes in the temperature by relocating themselves [31]. It has been also demonstrated that *N*. *littoralis* form a feeding matrix on carrion that is the complex microenvironment where the larvae feed and warm-up, as it also generates heat [22]. To form the optimal matrix, activity of many larvae is necessary. Moreover, the earlier presence of adult beetles on the meat improved the quality of the matrix, with a decrease in mortality and development time of larvae that stayed in the matrix [22]. Therefore, we expect that under individual rearing conditions the larvae will not form a typical feeding matrix, which will negatively affect their survival and development.

Considering the ecology of *N*. *littoralis* and assuming that food intake and temperature are crucial factors that affect growth of these insects, the question arises, whether separating larvae of *N*. *littoralis* significantly affects results of developmental research? In this study, we tested whether rearing of immature *N*. *littoralis* individually influences the quality of resultant development data in comparison to the data obtained in the communal rearing conditions. We hypothesized that: 1) separating larvae lowers their survival; 2) rearing them in aggregations accelerates development and 3) collective rearing increases the size of the beetles at maturity.

## Materials and methods

Adult beetles were taken from a laboratory colony established in 2017 using the beetles sampled in alder forest of Biedrusko military range (52°31’N, 16°54’E; Western Poland). The colony is maintained at Laboratory of Criminalistics at AMU (Poznań, Poland). Pairs of adult beetles were placed in 0.5 litre containers to allow them to lay eggs. They had pork *ad libitum* and constant access to cotton balls soaked with water. Containers were checked every 4 hours. The batches of eggs were transferred into 0.5 l containers filled with soil and placed in temperature chambers (ST 1/ 1 BASIC or ST 1+/ST 1+, POL-EKO, Poland) at five constant temperatures: 14, 15, 16, 20, and 26°C. Containers were inspected for the presence of fresh first instar larvae (creamy white, not fully sclerotized) at time intervals of about 10% of the egg stage duration, which was calculated for each temperature based on the pilot study. The larvae were reared in incubators at the same constant temperatures according to two different protocols: individual rearing and rearing in aggregations.

### Individual rearing

Thirty larvae per temperature were separated into 120 ml plastic containers with soil, cotton balls soaked with water (replenished during each inspection), and pieces (about 10 grams) of pork. New pieces of meat were added each time it had been found that the meat dried out or that there was less than half of its initial amount. Larvae were monitored in terms of transition to subsequent developmental stage (2nd instar larva, 3rd instar larva, post-feeding larva, pupa and adult beetle) at predetermined intervals, representing no more than 10% of the duration of the stage. Larval developmental stages were determined based on their colour, size and proportions of the body. Freshly moulted larvae are creamy-white and non-sclerotized. They darken with time, so the larvae that are shortly after ecdysis are easy to recognize. The size and proportions of the larvae also change very clearly during ecdysis, so we used them as supplementary features to distinguish larval instars. When 3rd instar larvae ceased feeding (they start to bury themselves at this moment), remains of meat were removed and the soil in the containers was added to allow formation of pupal chambers. Beetles remained in the containers until the immature development was completed. Body length was measured for all of the emerged adult beetles. Insects were put into a transparent vial, placed against a geometrical micrometer [32] and their body length (from the clypeus to the end of the last abdominal segment) was read when they were fully erect and immobile. Their body was weighed using an analytical balance (AS 82/220.R2, Radwag, Poland; readability = 0.01/0.1 mg, repeatability [5% Max] = 0.015 mg, linearity = ±0.06/±0.2mg).

### Rearing in aggregation

Four egg batches at each temperature were incubated. After larvae hatched, they were distributed at random into four small terrariums (30 x 20 x 20 cm) to allow them to form an aggregation (50 larvae per terrarium). In each terrarium, there was soil, cotton balls soaked with water, and pork *ad libitum* (pieces of about 80 grams, replenished if necessary). The larvae were inspected for developmental landmarks at the same time intervals as in the individual rearing protocol. The given landmark was reached when half of the larvae in the aggregation passed to the next developmental stage. The post-feeding larvae were transferred to 0.5 litre plastic containers (10 individuals per container) filled with soil to enable them to form pupal chambers. They remained there until emergence. Adult beetles were measured and weighed as in the individual protocol. Mortality of the post-feeding larvae and pupae was assessed at the end of the experiment. Containers were emptied and checked for the presence of dead beetles. Dead post-feeding larvae indicated that death occurred at the post-feeding stage, dead pupae indicated that death occurred at the pupal stage.

### Data analyses

We calculated the percentage mortality for both protocols at each of the analysed temperatures. By counting beetles after each developmental landmark (e.g. first ecdysis or pupation), we collected data on mortality at particular life stages. We compared mortality between beetles bred individually and in aggregations using the Wilcoxon signed-rank test. To analyse the influence of the protocol (individual or in aggregations) and the temperature on the duration of development, body length and weight of adult beetles, we used the two-way ANOVA. The Tukey’s HSD test was used for post hoc comparisons. All statistical analyses were made using Statistica 13 (TIBCO Software Inc.).

### Ethical approval

The study comprised laboratory experiments using insect species *Necrodes littoralis* (Coleoptera: Silphidae). The species is not under protection. No permission or approval from Ethic Commission were needed.

## Results

**Fig 1:**
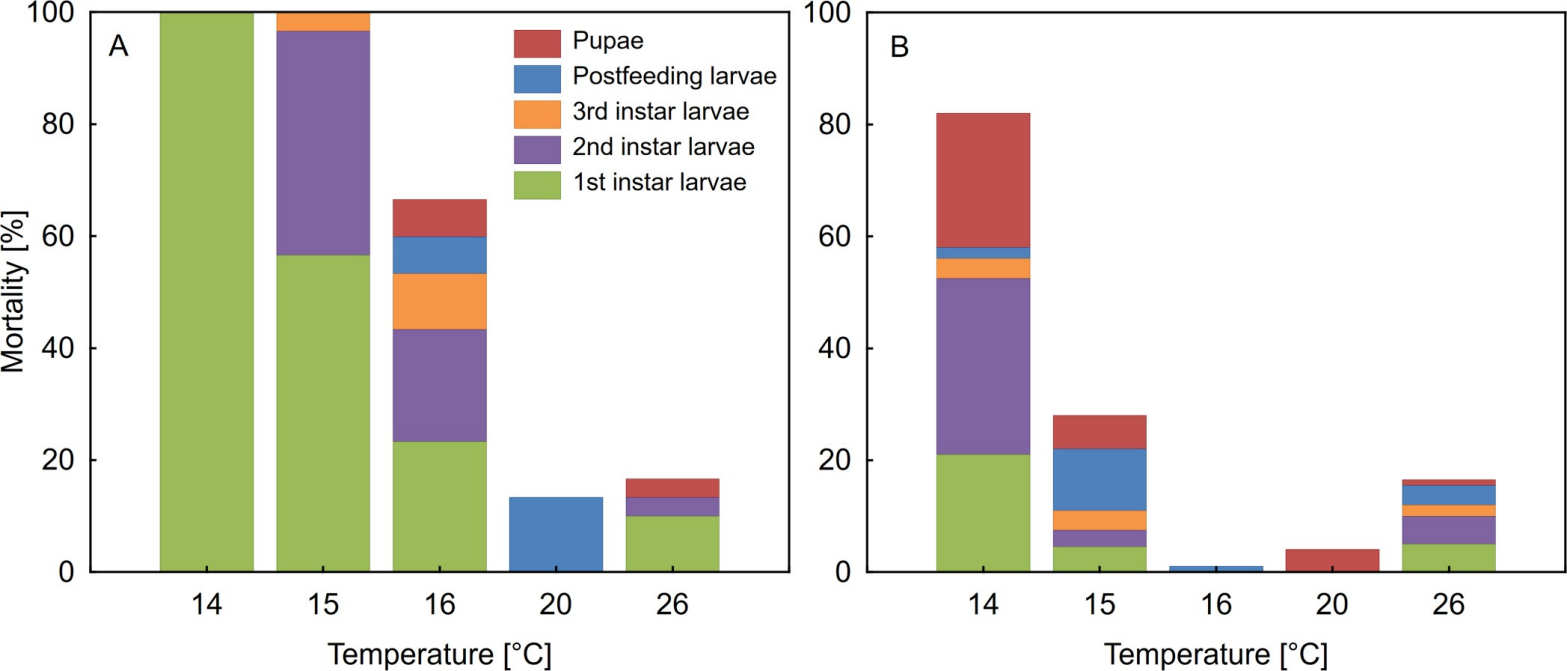
Mortality of *Necrodes littoralis* at preimaginal stages reared individually (A) and in aggregations (B).

There were significant differences in mortality between the rearing protocols (the Wilcoxon signed-rank test; *Z* = 2.02, *p* = 0.043, N = 5). At all temperatures mortality was much higher for insects reared individually (Fig 1). There was 100% mortality of the beetles reared individually at 14°C and 15°C. Differences were also observed between the protocols in the distribution of deaths across developmental stages (Fig 1). Larvae reared individually died more often at the early stages of development (1^st^ instar and 2^nd^ instar larva), while mortality of the collectively reared larvae was much lower during these stages. At the highest tested temperature (26°C), there was no influence of the laboratory protocol on the mortality (Fig 1).

Both the temperature and the protocol type affected the development time, length and weight of adult beetles (two-way ANOVA, *p*<0.05, Table 1). There was also a statistically significant interaction between the protocol type and the rearing temperature (Table 1).

**Table 1:** Results of ANOVA for effects of temperature and rearing method on the development duration, length and weight of adult *Necrodes littoralis*.

Dependent variable	Factors	df	F	p
Duration of development	Temperature	2	4780.66	< 0.001
	Protocol	1	41.41	< 0.001
	Temperature × Protocol	2	119.55	< 0.001
Body length	Temperature	2	45.96	< 0.001
	Protocol	1	61.71	< 0.001
	Temperature × Protocol	2	9.57	< 0.001
Body weight	Temperature	2	44.38	< 0.001
	Protocol	1	5.19	0.023
	Temperature × Protocol	2	13.78	< 0.001

**Fig 2:**
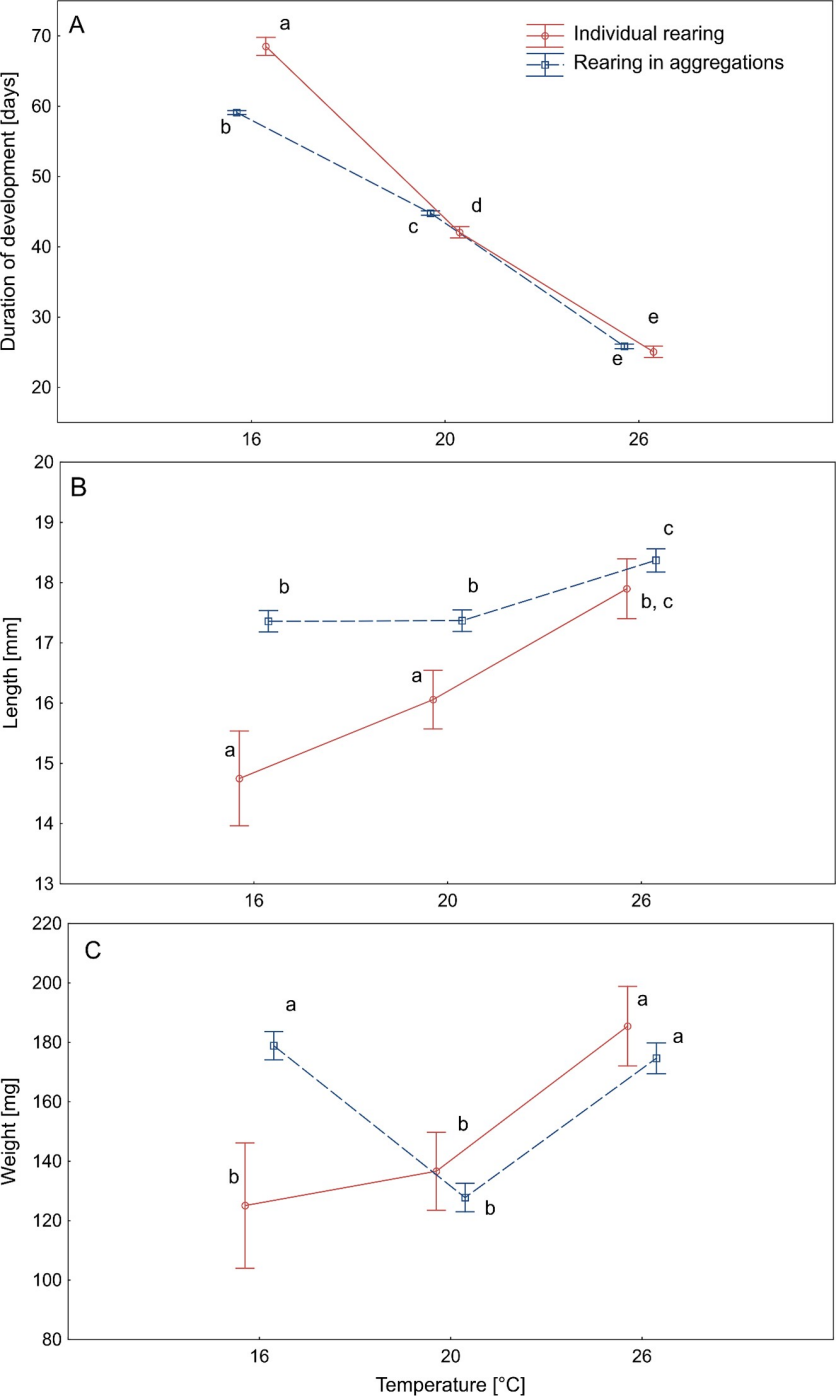
Mean duration of development (A), length (B) and weight (C) of adult *Necrodes littoralis* reared individually and in aggregations. Duration of development is time in days from the oviposition to the emergence of an adult beetle. Whiskers represent the 0.95 confidence intervals. Different letters denote significant differences in pairwise comparisons at *p* = 0.05 using Tukey’s HSD test. We used all emerged individuals for the calculations. The total sample size at 16°C, 20°C and 26°C was respectively: 198, 192 and 167 in the protocol with aggregations and 10, 26 and 25 in the protocol with individual rearing.

Statistically significant differences between the protocols in the duration of development were recorded at 16°C (Tukey’s HSD: *p*<0.001) and 20°C (Tukey’s HSD: *p*<0.001) (Fig 2A). At 16°C individually reared specimens developed 15.9% longer than those reared in aggregations (Fig 2A). At 20°C, development of the beetles reared individually was shorter by 6.1% than in aggregations (Fig 2A). Beetles that developed in aggregations at 16°C (Tukey’s HSD: *p*<0.001) and 20°C (Tukey’s HSD: *p* = 0.003) were significantly longer than those reared individually (Fig 2B). Significant differences in body weight between the protocols were recorded only at 16°C (Tukey’s HSD: *p* = 0.005), where the mass of adult beetles reared in aggregations was much larger than under individual rearing conditions (Fig 2C).

## Discussion

The individual rearing protocol excluded collection of full data on the duration and size of adult *N*. *littoralis* at low temperatures (14 and 15°C). Rearing in aggregations at the lowest temperature (14°C) was also associated with high mortality. However, it allowed for the collection of the complete data for a relatively large number of beetles. These results revealed a similar temperature pattern as in the previous studies of necrophilous beetles, i.e. a very high mortality at low temperatures [9,11,13]. This can be a problem when multiple temperatures need to be studied to create a developmental model. Rearing larvae in aggregations provided low-temperature data in case of *N*. *littoralis* and we believe that in other forensically-important gregarious beetles, this protocol may be similarly effective. Study by Scanvion et al. [20] showed a decrease in mortality with an increase in larval density of *Lucilia sericata* (Meigen, 1826) (Diptera: Calliphoridae). In *C*. *megacephala* and *C*. *rufifacies* (Macquart, 1842) (Diptera: Calliphoridae), an increase in density similarly reduced the mortality, but only up to a point, where the further increase in larval density had an adverse effect [24]. Current results indicate that this relationship may be true not only for blow flies but also for gregarious carrion beetles.

Results for the time of development at 16°C suggest that the relationship between density and development of carrion beetles may be similar to the one found previously for carrion blow flies. In *Calliphora vicina* Robineau-Desvoidy, 1830 (Diptera: Calliphoridae), the greater the density of larvae in the aggregation, the faster the development [23]. Also in *C*. *megacephala* and *C*. *rufifaces*, density had a positive effect on the rate of development in the first and the second instar larvae [24]. In *P*. *regina*, rearing the larvae in aggregations of 10 insects shortened the development time [33]. In *L*. *sericata* development time also depended on density, but the decrease was observed only for densities from 50 to 250 larvae [20]. Sullivan and Sokal [34] studied the effect of seven rearing densities on the body mass of four *Musca domestica* Linnaeus, 1758 (Diptera: Muscidae) strains. Although the increase in density, in general, had a negative effect on body mass, this was only significant at high densities. Depending on the strain, at the lowest densities, the decrease in mass was insignificant or the body mass even increased. Only in groups larger than 160 individuals, there was a visible decrease in body mass. In *P*. *regina*, rearing the larvae in aggregations of 10 individuals had a significant, positive effect on the mass of the puparia [33].

The maximum body length in adult *Necrodes littoralis* collected from the field can reach around 26 mm [35]. Beetles in our study were systematically smaller than those developing under natural conditions. Probably the laboratory conditions used in this study were suboptimal for the species. The reason may be the absence of adult beetles on the meat in the pre-larval phase. Other studies have shown that the earlier presence of adult *Necrodes* beetles on meat can have a positive effect on the body mass of developing larvae [22]. It is also possible that other factors, such as humidity or the quality and quantity of larval diet may affect the final size of the beetles. Further studies are necessary on this topic. Moreover, at 20°C, rearing of the larvae in aggregations resulted in a significant decrease in their mass, but not length compared to the other temperatures. This was probably due to the malnutrition of the third instar larvae at the end of the stage, when the body length does not change but the larvae are gaining mass. Due to unknown reasons these larvae might have ceased feeding too early.

Because at the highest temperature there were no significant differences in mortality, development time and size of adult beetles between the rearing protocols, it seems that high temperatures somehow eliminate negative effects of individual rearing. Bacterial degradation of meat may be of importance here. The activity of microbes causes meat to putrefy that partially disintegrates its structure and makes it easier for the beetle larvae to consume. High temperatures enhance the activity of microbes and putrefaction that makes it easier for the beetle larvae to consume meat. At low temperatures, putrefaction is very limited and therefore the ‘unprocessed’ meat may be difficult for a single larva to consume. Collective feeding can facilitate food intake by the larvae under such temperatures. Previous studies on necrophagous flies supported the hypothesis of digestion benefits from the collective feeding by the larvae [19]. Results for *L*. *sericata* indicated that collective exodigestion can be beneficial for the larvae, as the experimental enrichment of the food with digestive enzymes reduced the time of development under low larval densities [20]. A similar mechanism may occur in *N*. *littoralis*. It was found that larvae of *N*. *littoralis* apply their exudates to the food substrate, forming a feeding matrix on carrion [22] This microenvironment may be formed only by an aggregation of larvae. As a result, meat does not dry and is more accessible for the larvae. Moreover, the feeding matrix produces heat that brings extra benefits for the aggregated larvae [22]. A similar aggregation of larvae and formation of the feeding matrix was observed in *Diamesus osculans* (Vigors, 1825), a closely related species, distributed from India to Australia (J. Růžička, personal communication). This is probably related to the similar ecology of the species and reproduction on large carrion. This indicates that results from this study may be important not only for *Necrodes* beetles but also for other closely related carrion beetles (e.g. *Diamesus* Hope, 1840; *Oxelytrum* Gistel, 1848 or *Ptomaphila* Kirby & Spence, 1828). Studies on other feeding protocols, e.g. with the presence of adult insects on the meat before providing it to the larvae, could further improve the rearing protocols for gregarious carrion beetles.

More research is also needed on the size of an aggregation and its impact on the development of *N*. *littoralis*. We examined the larval development in two densities only; therefore further studies are necessary to identify benefits of the larger aggregations. Nevertheless, even in small aggregations, as demonstrated in this study, the effect of aggregating on development of larvae was clear, especially at low temperatures. These effects are important not only for the development research in forensic entomology but also for the estimation of PMI in forensic investigations. If larvae of *N*. *littoralis* from a death scene are reared in the laboratory under individual conditions, the mortality may be high especially at low temperatures. Therefore, in a forensic practice we recommend rearing the larvae of *N*. *littoralis* in the aggregations. If the number of larvae collected on a death scene is low, they should be reared at high temperatures (preferably over 20°C), as it may prevent the loss of insect evidence due to an increased mortality. Another way to facilitate rearing of small number of larvae from a death scene would be to use the previously prepared food substrate. Placing adult beetles or other *Necrodes* larvae on the meat to be used as a food substrate will provide a feeding matrix [22], which may enhance rearing success for the death scene insects. Unfortunately, the laboratory colony of *Necrodes* beetles is necessary for this purpose. Also, deriving thermal summation models using data from the individual rearing conditions can lead to errors in PMI estimation, as the physiological age (*K*) of death scene insects would be overestimated using such models. Consequently, the minimum PMI would be also overestimated, especially in spring and early summer cases, when the temperatures are frequently much below 20°C [36]. A similar problem may occur when creating an isomegalen diagram (a graph modelling the size of the larvae in relation to their age and temperature) [37]. Individually reared larvae will be smaller than those developing in aggregations under natural conditions. Therefore, if one uses data from individual rearing protocol to create isomegalen diagram, and then on its basis to estimate the age of the larvae from a death scene (which are larger due to the benefits of aggregation), the result will be an overestimation of insect age. Similarly, the individual rearing protocol may limit the use of linear regression models between physiological age of insects and their size at maturity. Such models may improve the accuracy of PMI estimation as they allow for the calibration of physiological age for insects sampled on a death scene [25,38].

## Conclusions

This study demonstrated that aggregation is beneficial for larvae of *N*. *littoralis*, resulting in lower mortality, shorter development time, and larger size. These effects depend on the temperature experienced by the larvae during development and occur primarily at low temperatures. Therefore, in gregarious carrion beetles they need to be considered, both while designing the developmental research for forensic applications and while rearing larvae sampled on a death scene in criminal investigations. Moreover, further research is necessary to fully understand the relation between the aggregation behaviour of carrion beetle larvae, its developmental benefits and the temperature.

## Supporting information

S1 File Mortality data. This file contains data on the mortality of *N*. *littoralis* at different developmental stages. Values are expressed as a percentage.
(XLSX)

S2 File Development duration, length and weight data. This file contains data on the development time, body length and weight of adult *N*. *littoralis* within the two protocols.
(XLSX)

## References

[1] Amendt J, Campobasso CP, Gaudry E, Reiter C, LeBlanc HN, Hall MJR. Best practice in forensic entomology—standards and guidelines. International Journal of Legal Medicine. 2007;121:90–104. doi: 10.1007/s00414-006-0086-x 16633812

[2] Matuszewski S. Estimating the pre-appearance interval from temperature in *Necrodes littoralis* L. (Coleoptera: Silphidae). Forensic Science International. 2011;212(1–3):180–8. doi: 10.1016/j.forsciint.2011.06.010 21715113

[3] Catts EP. Problems in Estimating the Postmortem Interval in Death Investigations. Journal of Agricultural Entomology. 1992;9(4):245–55.

[4] Richards CS, Villet MH. Data quality in thermal summation development models for forensically important blowflies. Medical and Veterinary Entomology. 2009;23(3):269–76. doi: 10.1111/j.1365-2915.2009.00819.x 19712157

[5] Byrd JH, Allen JC. The development of the black blow fly, *Phormia regina* (Meigen). Forensic Science International. 2001;120(1–2):79–88. doi: 10.1016/s0379-0738(01)00431-5 11457615

[6] Byrd JH, Butler JF. Effects of Temperature on *Chrysomya rufifacies* (Diptera: Calliphoridae) Development. Journal of Medical Entomology. 1997;34(3):353–8. doi: 10.1093/jmedent/34.3.353 9151502

[7] Grassberger M, Reiter C. Effect of temperature on *Lucilia sericata* (Diptera: Calliphoridae) development with special reference to the isomegalen- and isomorphen-diagram. Forensic Science International. 2001;120(1–2):32–6. doi: 10.1016/s0379-0738(01)00413-3 11457606

[8] Voss SCC, David F., Hung W-F, Dadour IR. Survival and development of the forensically important blow fly, *Calliphora varifrons* (Diptera: Calliphoridae) at constant temperatures. Forensic Science, Medicine, and Pathology. 2014;10(3):314–21. doi: 10.1007/s12024-014-9565-4 24771477

[9] Frątczak-Łagiewska K, Grzywacz A, Matuszewski S. Development and validation of forensically useful growth models for Central European population of *Creophilus maxillosus* L. (Coleoptera: Staphylinidae). International Journal of Legal Medicine. 2020. doi: 10.1007/s00414-020-02275-3 32266535 PMC7295842

[10] Lambiase S, Murgia G, Sacchi R, Ghitti M, di Lucia V. Effects of Different Temperatures on the Development of *Dermestes frischii* and *Dermestes undulatus* (Coleoptera, Dermestidae): Comparison Between Species. Journal of Forensic Sciences. 2017;63(2):469–73. doi: 10.1111/1556-4029.13580 28631278

[11] Midgley JM, Villet MH. Development of *Thanatophilus micans* (Fabricius 1794) (Coleoptera: Silphidae) at constant temperatures. International Journal of Legal Medicine. 2009;123:103–8. doi: 10.1007/s00414-008-0260-4 18779975

[12] Ridgeway JA, Midgley JM, Collett IJ, Villet MH. Advantages of using developmental models of the carrion beetles *Thanatophilus micans* (Fabricius), and *T. mutilatus* (Castelneau) (Coleoptera: Silphidae) for estimating minimum post mortem intervals, verified with case data. International Journal of Legal Medicine. 2014;128:207–20. doi: 10.1007/s00414-013-0865-0 23974525

[13] Zanetti NI, Visciarelli EC, Centeno ND. The Effect of Temperature and Laboratory Rearing Conditions on the Development of *Dermestes maculatus* (Coleoptera: Dermestidae). Journal of Forensic Sciences. 2016;61(2):375–81. doi: 10.1111/1556-4029.12965 26477981

[14] Montoya-Molina S, Jakubec P, Qubaiová J, Novák M, Šuláková H, Růžička J. Developmental Models of the Forensically Important Carrion Beetle, *Thanatophilus sinuatus* (Coleoptera: Silphidae). Journal of medical entomology. 2020;tjaa255:1–7. doi: 10.1093/jme/tjaa255 33200199

[15] Velásquez Y, Viloria AL. Effects of temperature on the development of the Neotropical carrion beetle *Oxelytrum discicolle* (Brullé, 1840) (Coleoptera: Silphidae). Forensic Science International. 2009;185(1–3):107–9. doi: 10.1016/j.forsciint.2008.12.020 19201556

[16] Wang Y, Wang M, Hu G, Xu W, Wang Y, Wang J. Temperature-dependent development of *Omosita colon* at constant temperature and its implication for PMI min estimation. Journal of forensic and legal medicine. 2020;72(101946):101946. doi: 10.1016/j.jflm.2020.101946 32275229

[17] Martín-Vega D, Díaz-Aranda LM, Baz A, Cifrián B. Effect of Temperature on the Survival and Development of Three Forensically Relevant *Dermestes* Species (Coleoptera: Dermestidae). Journal of Medical Entomology. 2017;54(5):1140–50. doi: 10.1093/jme/tjx110 28549176

[18] Hu G, Wang M, Wang Y, Tang H, Chen R, Zhang Y, et al. Development of *Necrobia rufipes* (De Geer, 1775) (Coleoptera: Cleridae) under constant temperatures and its implication in forensic entomology. Forensic Science International. 2020;311:110275. doi: 10.1016/j.forsciint.2020.110275 32279028

[19] Rivers DB, Thompson C, Brogan RS. Physiological trade-offs of forming maggot masses by necrophagous flies on vertebrate carrion. Bulletin of entomological research. 2011;101(5):599–611. doi: 10.1017/S0007485311000241 21729395

[20] Scanvion Q, Hedouin V, Charabidze D. Collective exodigestion favours blowfly colonization anddevelopment on fresh carcasses. Animal Behaviour. 2018;141:221–32. doi: 10.1016/j.anbehav.2018.05.012

[21] Greenberg B. Flies as forensic indicators. Journal of Medical Entomology. 1991;28(5):565–77. doi: 10.1093/jmedent/28.5.565 1941921

[22] Matuszewski S, Mądra-Bielewicz A. Heat production in a feeding matrix formed on carrion by communally breeding beetles. Frontiers in Zoology. 2021;18(5(2021)). doi: 10.1186/s12983-020-00385-7 33526056 PMC7851950

[23] Saunders D. Effects of larval crowding on size and fecundity of the blow fly, *Calliphora vicina* (Diptera: Calliphoridae). European Journal of Entomology. 1995;92(4):615–22.

[24] Goodbrod JR, Goff ML. Effects of larval population density on rates of development and interactions between two species of *Chrysomya* (Diptera: Calliphoridae) in laboratory culture. Journal of medical entomology. 1990;27(3):338–43. doi: 10.1093/jmedent/27.3.338 2332878

[25] Gruszka J, Matuszewski S. Estimation of physiological age at emergence based on traits of the forensically useful adult carrion beetle *Necrodes littoralis* L. (Silphidae). Forensic Science International. 2020;314:110407. doi: 10.1016/j.forsciint.2020.110407 32673947

[26] Matuszewski S, Bajerlein D, Konwerski S, Szpila K. Insect succession and carrion decomposition in selected forests of Central Europe. Part 1: Pattern and rate of decomposition. Forensic Science International. 2010;194(1–3):85–93. doi: 10.1016/j.forsciint.2009.10.016 19914786

[27] Matuszewski S, Bajerlein D, Konwerski S, Szpila K. Insect succession and carrion decomposition in selected forests of Central Europe. Part 2: Composition and residency patterns of carrion fauna. Forensic Science International. 2010;195(1–3):42–51. doi: 10.1016/j.forsciint.2009.11.007 20018471

[28] Charabidze D, Vincent B, Pasquerault T, Hedouin V. The biology and ecology of *Necrodes littoralis*, a species of forensic interest in Europe. International Journal of Legal Medicine. 2016;130:273–80. doi: 10.1007/s00414-015-1253-8 26762393

[29] Ratcliffe BC. The natural history of *Necrodes surinamensis* (FABR.)(Coleoptera: Silphidae). Transactions of the American Entomological Society. 1972;98(4):359–410.

[30] Frątczak K, Matuszewski S. Instar determination in forensically useful beetles *Necrodes littoralis* (Silphidae) and *Creophilus maxillosus* (Staphylinidae). Forensic science international. 2014;241:20–6. doi: 10.1016/j.forsciint.2014.04.026 24835031

[31] Gruszka J, Krystkowiak-Kowalska M, Frątczak-Łagiewska K, Mądra-Bielewicz A, Charabidze D, Matuszewski S. Patterns and mechanisms for larval aggregation in carrion beetle *Necrodes littoralis* (Coleoptera: Silphidae). Animal Behaviour. 2020;162:1–10. doi: 10.1016/j.anbehav.2020.01.011

[32] Villet MH. An inexpensive geometrical micrometer for measuring small, live insects quickly without harming them. Entomologia Experimentalis et Applicata. 2007;122(3):279–80. doi: 10.1111/j.1570-7458.2006.00520.x

[33] Green PWC, Simmonds MSJ, Blaney WM. Diet nutriment and rearing density affect the growth of black blowfly larvae, *Phormia regina* (Diptera: Calliphoridae). European journal of entomology. 2003;100(1):39–42. doi: 10.14411/eje.2003.008

[34] Sullivan RL, Sokal RR. The effects of larval density on several strains of the house fly. Ecology. 1963;44(1):120–30. doi: 10.2307/1933186

[35] Šustek Z. Key to identification of insects: carrion beetles of Czechoslovakia (Coleoptera, Silphidae). Zprávy Československé Společnosti Entomologické při ČSAV, Klíče k určování hmyzu. 1981;2:1–47.

[36] Michalski M, Nadolski J. Thermal conditions in selected urban and semi-natural habitats, important for the forensic entomology. Forensic Science International. 2018;287:153–62. doi: 10.1016/j.forsciint.2018.03.042 29665482

[37] Amendt J, Richards CS, Campobasso CP, Zehner R, Hall MJR. Forensic entomology: applications and limitations. Forensic Sci Med Pathol. 2011;7(4):379–92. doi: 10.1007/s12024-010-9209-2 21213072

[38] Matuszewski S, Frątczak-Łagiewska K. Size at emergence improves accuracy of age estimates in forensically-useful beetle *Creophilus maxillosus* L.(Staphylinidae). Scientific reports. 2018;8(1):1–9. doi: 10.1038/s41598-017-17765-5 29402934 PMC5799346

